# Supplementary figures and images for: The transcriptional response in mosquitoes distinguishes between fungi and bacteria but not Gram types
Source: BMC Genomics. 2024 Apr 9;25:353. doi: 10.1186/s12864-024-10153-0 (PMC11003161; doi:10.1186/s12864-024-10153-0)

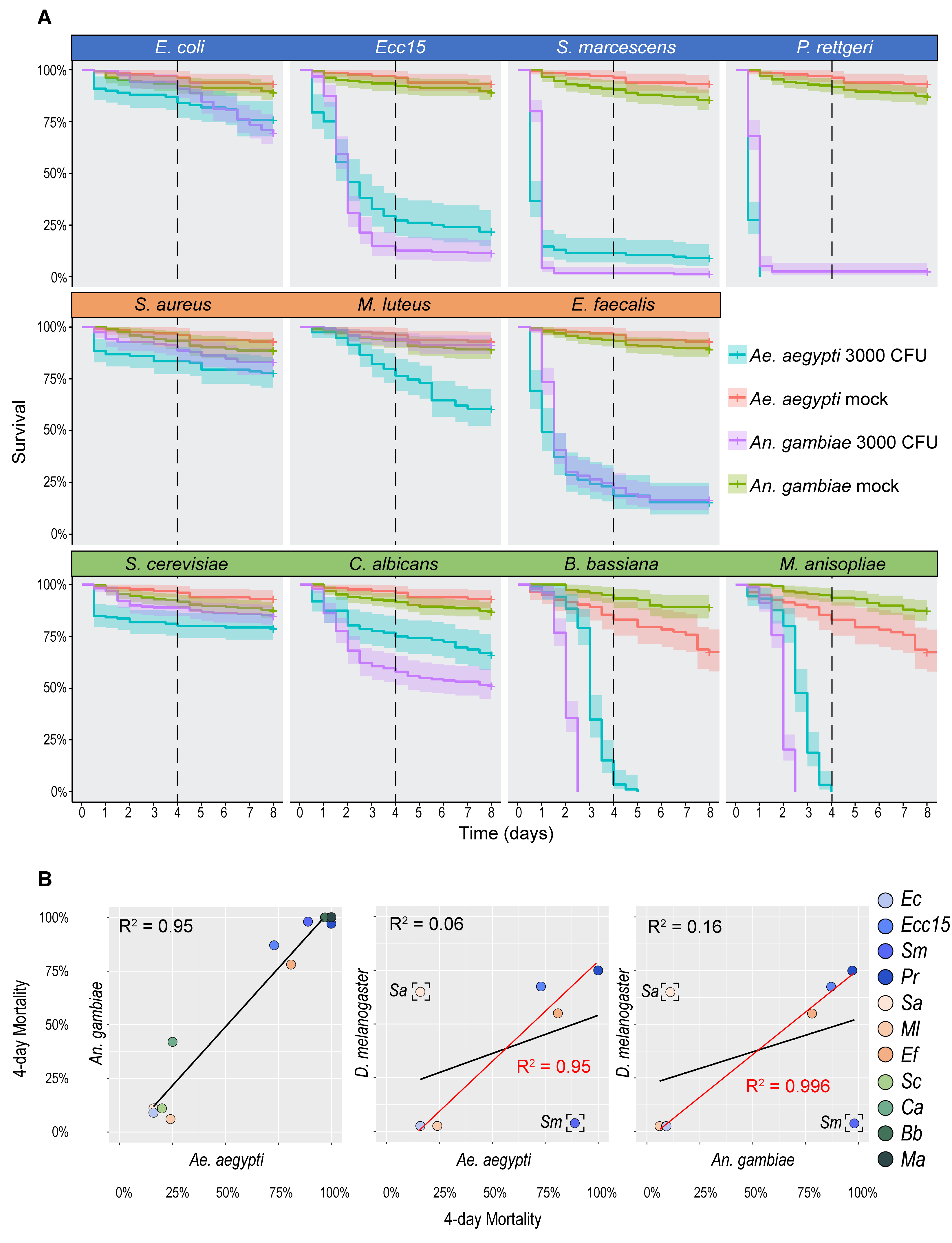

Supplement: Supplementary file 1 — Additional file 1: Fig. 1 S1. Mortality following systemic infection with bacteria, yeasts, and filamentous fungi is closely correlated in Aedes aegypti and Anopheles gambiae. (A) Mosquitoes were injected with 3000 colony-forming units or conidia of Gram-negative bacteria (blue row), Gram-positive bacteria (orange row), and fungal (green row) pathogens. Pathogens include Escherichia coli (Ec), Erwinia carotovora carotovora 15 (Ecc15), Serratia marcescens type strain (Sm), Providencia rettgeri (Pr), Staphylococcus aureus (Sa), Micrococcus luteus (Ml), Enterococcus faecalis (Ef), Saccharomyces cerevisiae (Sc), Candida albicans (Ca), Beauveria bassiana (Bb), and Metarhizium anisopliae (Ma). Dotted lines mark 4 days post-infection; in this study, mortality at 4 days serves as a proxy value for pathogen virulence in a host. (B) Correlations of mortality of all pathogens in Ae. aegypti versus An. gambiae (s.l.), Ae. aegypti versus Drosophila melanogaster, and An. gambiae versus D. melanogaster. Bracketed data points are included in the linear regressions shown in black, but censored from the linear regressions shown in red. [file 12864_2024_10153_MOESM1_ESM.tif]

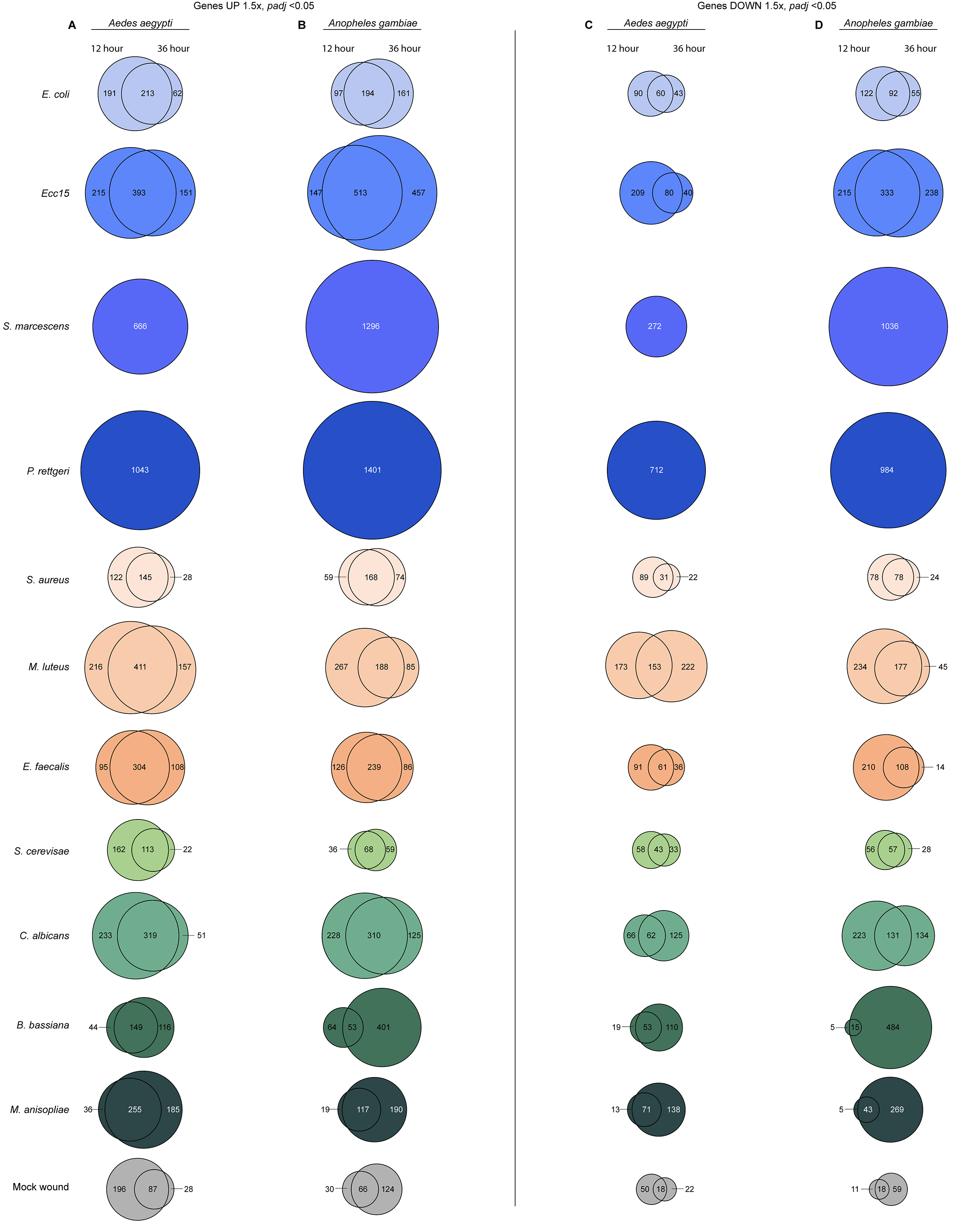

Supplement: Supplementary file 2 — Additional file 2: Fig. 2 S1. Differentially expressed genes in Aedes aegypti and Anopheles gambiae at 12 versus 36 hours post-infection. Venn diagrams displaying the number of genes upregulated (left) and downregulated (right) at 12 hours versus 36 hours post-challenge in Ae. aegypti (A, C) and An. gambiae (s.l.) (B, D) mosquitoes. Challenges included mock wounding and live infection with 3000 CFUs of Escherichia coli, Erwinia carotovora carotovora 15 (Ecc15), Serratia marcescens type strain, Providencia rettgeri, Staphylococcus aureus, Micrococcus luteus, Enterococcus faecalis, Saccharomyces cerevisiae, Candida albicans, and 3000 conidia of Beauveria bassiana, and Metarhizium anisopliae. Transcriptomes were assayed by RNAseq at 12 and 36 hours post-challenge, except where mortality was too high to collect the later timepoint (S. marcescens and P. rettgeri). Criteria for differential expression are ≥1.5x fold-change (up) or − 1.5x fold-change (down) relative to unchallenged, and padj < 0.05, as calculated by DESeq2. [file 12864_2024_10153_MOESM2_ESM.tif]

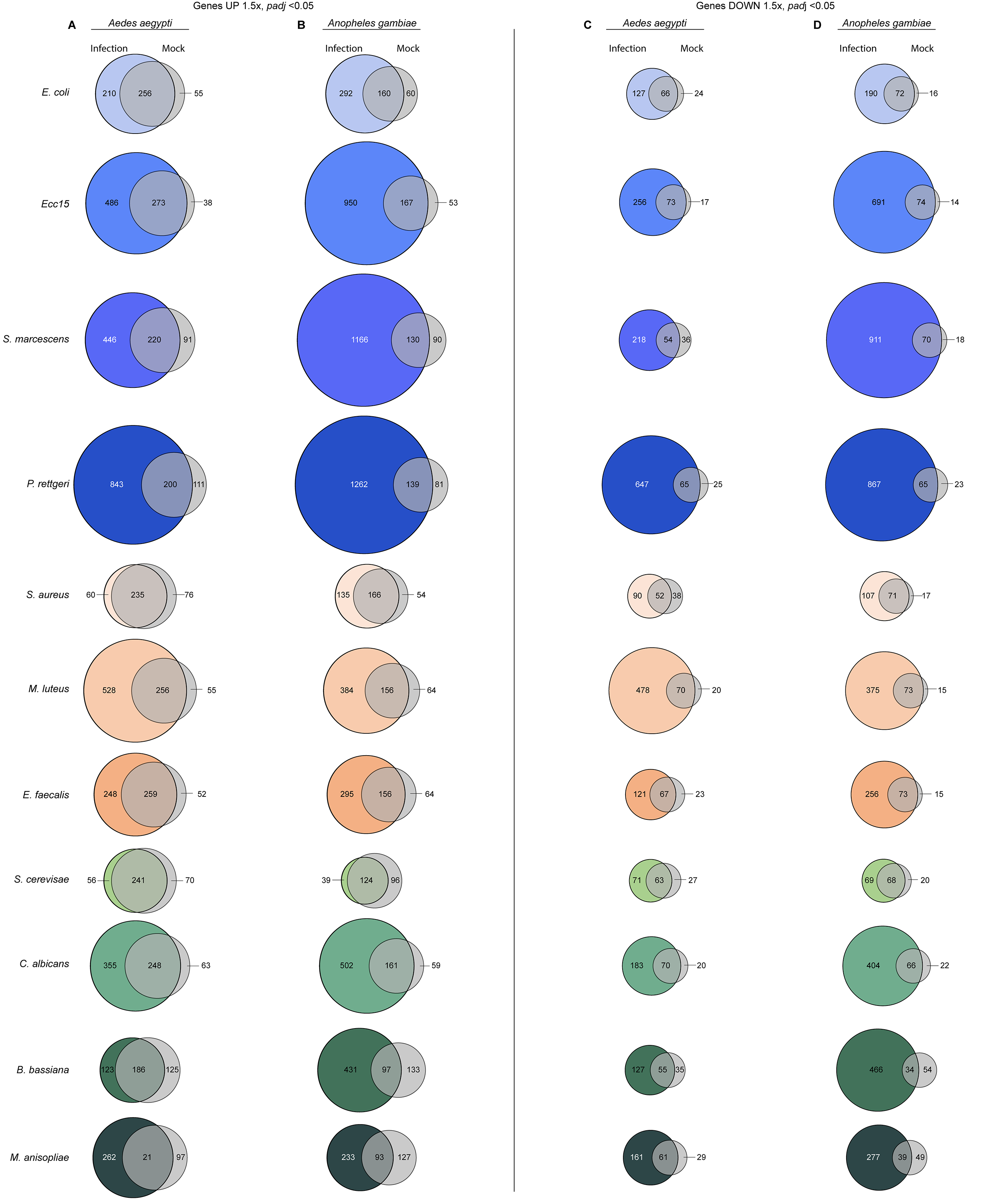

Supplement: Supplementary file 3 — Additional file 3: Fig. 2 S2. Differentially expressed genes in Aedes aegypti and Anopheles gambiae, infection versus mock wounding. Venn diagrams display the number of genes upregulated (left) and downregulated (right) in infected Ae. aegypti (A, C) and An. gambiae (s.l.) (B, D) mosquitoes versus mock-wounded counterparts. Live challenges included infection with 3000 CFUs of Escherichia coli, Erwinia carotovora carotovora 15 (Ecc15), Serratia marcescens type strain, Providencia rettgeri, Staphylococcus aureus, Micrococcus luteus, Enterococcus faecalis, Saccharomyces cerevisiae, Candida albicans, and 3000 conidia of Beauveria bassiana, and Metarhizium anisopliae. Transcriptomes were assayed by RNAseq at 12 and 36 hours post-challenge, except where mortality was too high to collect the later timepoint (S. marcescens and P. rettgeri). The count of regulated genes per condition is inclusive of both timepoints. Criteria for differential expression are ≥1.5x fold-change (up) or − 1.5x fold-change (down) relative to unchallenged, and padj < 0.05, as calculated by DESeq2. [file 12864_2024_10153_MOESM3_ESM.tif]

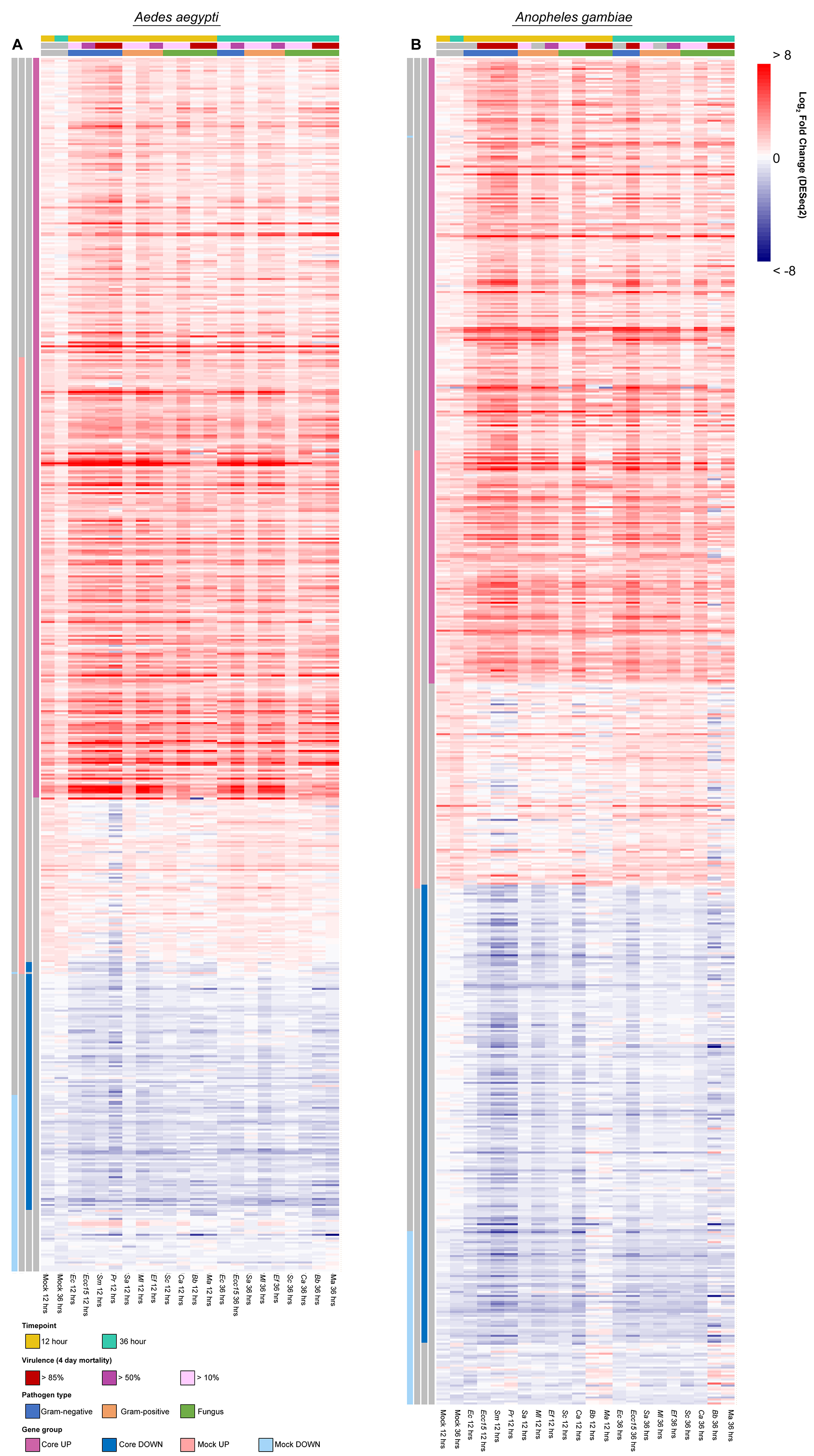

Supplement: Supplementary file 4 — Additional file 4: Fig. 2 S3. The amplitude of change in pan-microbial core genes is greater following live infection compared with mock wounding. Unscaled heatmaps of fold change (relative to unchallenged, calculated by DESeq2) in mock-infected and live-infected conditions at 12 and 36 hour post-challenge in Aedes aegypti (A) and Anopheles gambiae (s.l.) (B) mosquitoes, including all genes from the following groups: UP core genes, DOWN core genes, and genes that were differentially expressed (at either timepoint) following mock infection. Criteria for differential expression are ≥1.5x fold-change (up) or − 1.5x fold-change (down) relative to unchallenged, and padj < 0.05, as calculated by DESeq2. [file 12864_2024_10153_MOESM4_ESM.tif]

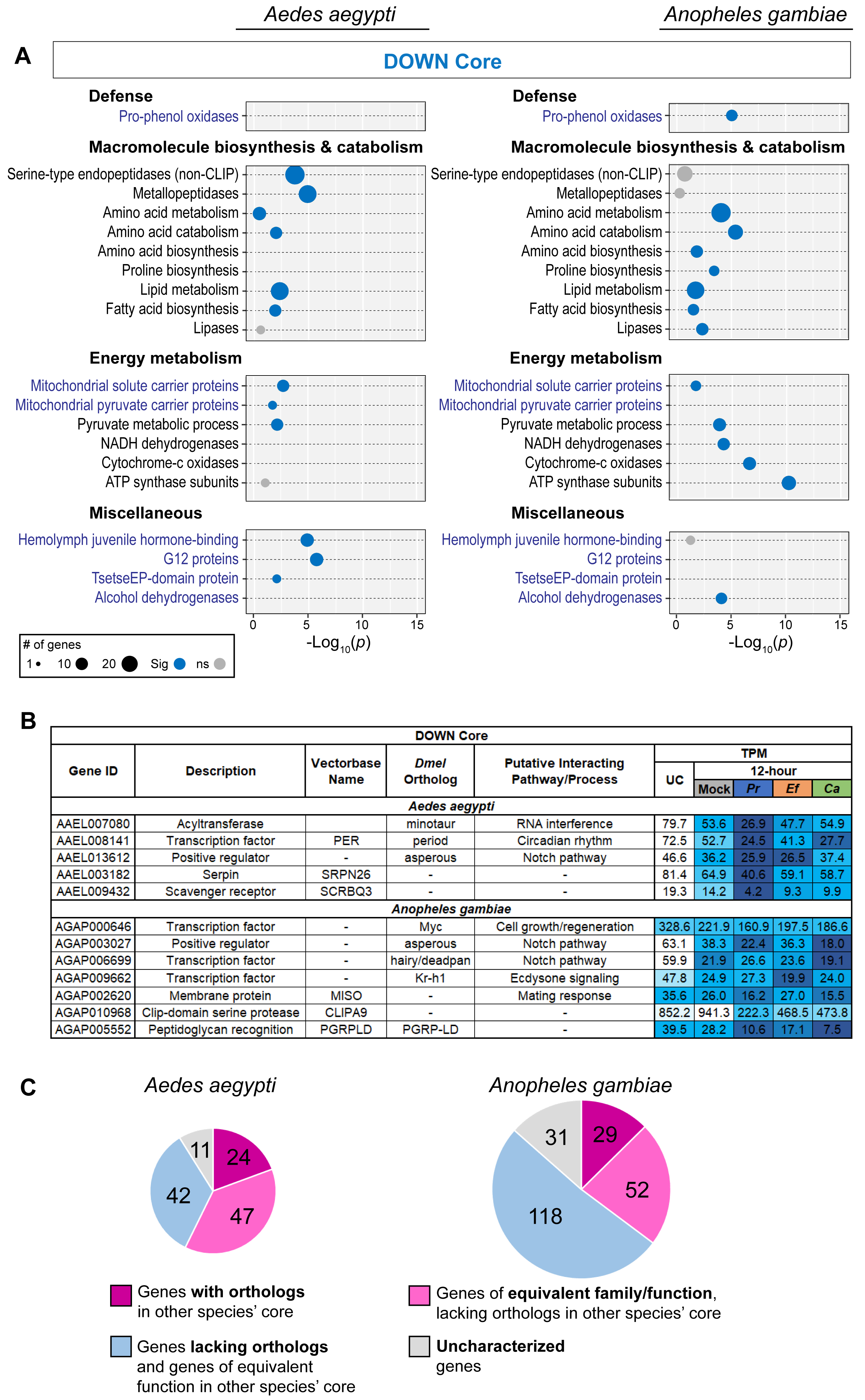

Supplement: Supplementary file 5 — Additional file 5: Fig. 3 S1. The downregulated core response to infection in Aedes aegypti and Anopheles gambiae. Bubble plots (A) display categories of genes enriched in the downregulated pan-microbial cores of Ae. aegypti and/or An. gambiae. Black text indicates category is from TopGO. Blue text indicates custom category (either from immune gene list, or defined by the presence of InterPro domain(s)). The size of the bubble is proportional to the number of genes from the given category in the downregulated core. The placement of the bubble along the x axis corresponds to the statistical significance of the enrichment (Fisher’s exact test). Gray bubbles indicate p value ≥0.05 (not significant enrichment). (B) Table of selected genes in the downregulated cores of Ae. aegypti and An. gambiae. (C) Pie charts describing the orthology and functional similarities shared by the Ae. aegypti and An. gambiae downregulated cores. [file 12864_2024_10153_MOESM5_ESM.tif]

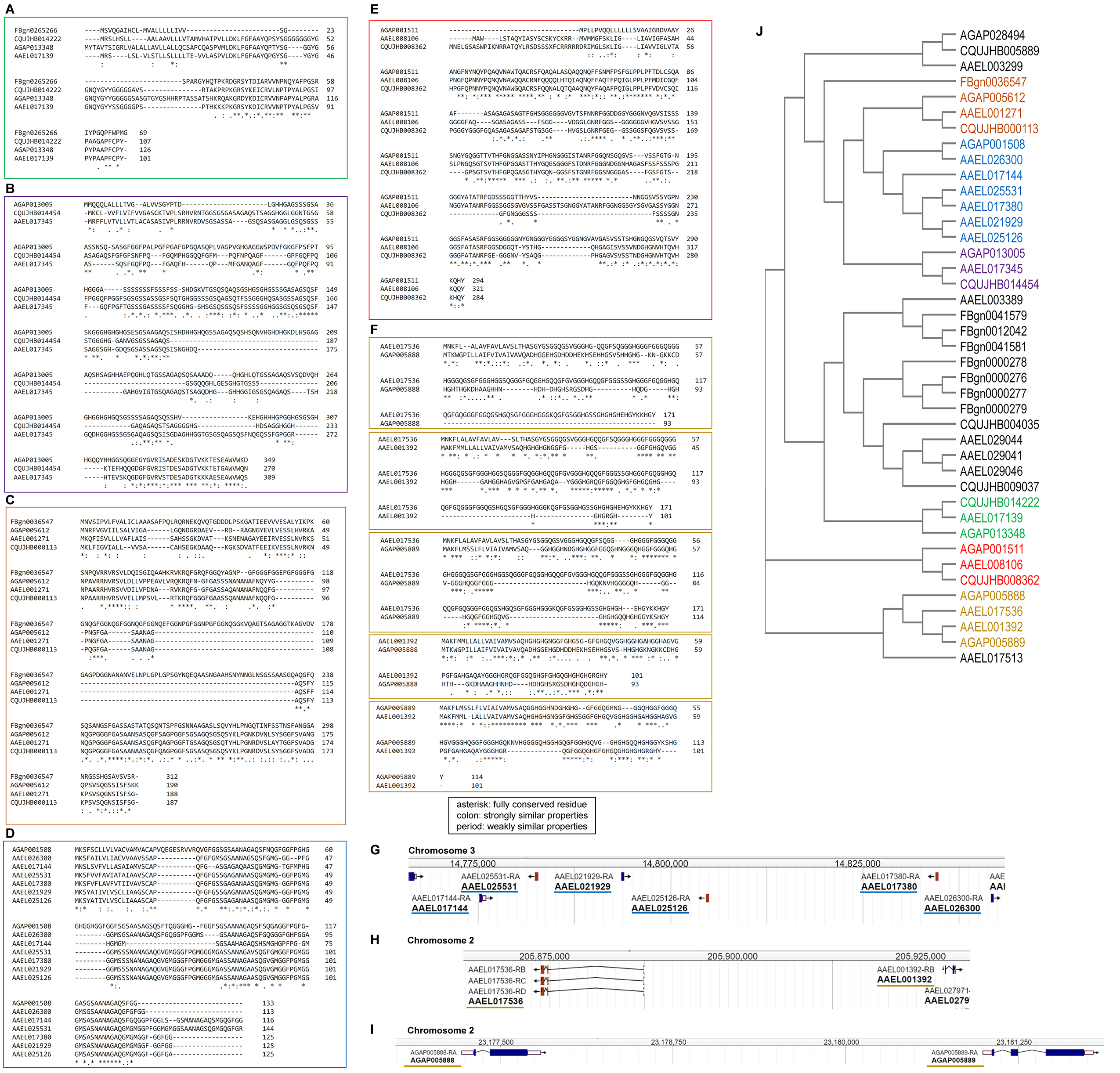

Supplement: Supplementary file 6 — Additional file 6: Fig. 3 S2. Infection-responsive glycine-rich proteins in Aedes aegypti and Anopheles gambiae share sequence similarity with each other, and with genes from Culex quinquefasciatus and Drosophila melanogaster. (A-F) Sequence alignments of glycine-rich (≥13%) proteins by Clustal Omega. (G-I) Genomic locations of glycine-rich proteins of clusters D and F in Ae. aegypti and An. gambiae. Images are derived from VectorBase Genome Browser. (J) A dendrogram of glycine-rich proteins from the Ae. aegypti and An. gambiae cores, together with related proteins from Drosophila melanogaster and Culex quinquefasciatus, generated by Clustal Omega. [file 12864_2024_10153_MOESM6_ESM.tif]

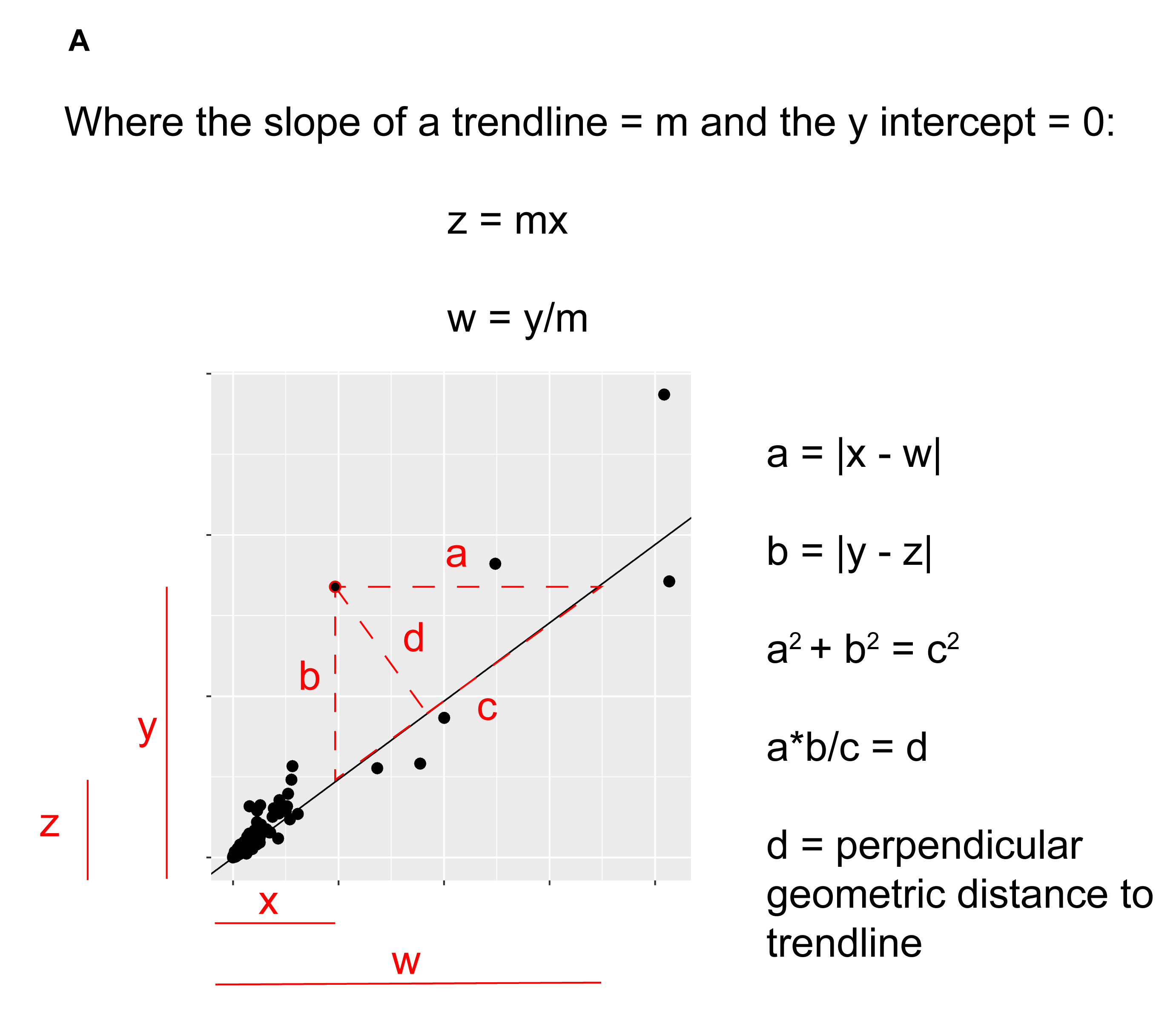

Supplement: Supplementary file 9 — Additional file 9: Fig. 4 S1. Calculation of the geometric perpendicular distance of a data point from a trendline. Method for deriving geometric distances using x and y values and the slope of a linear regression. [file 12864_2024_10153_MOESM9_ESM.tif]

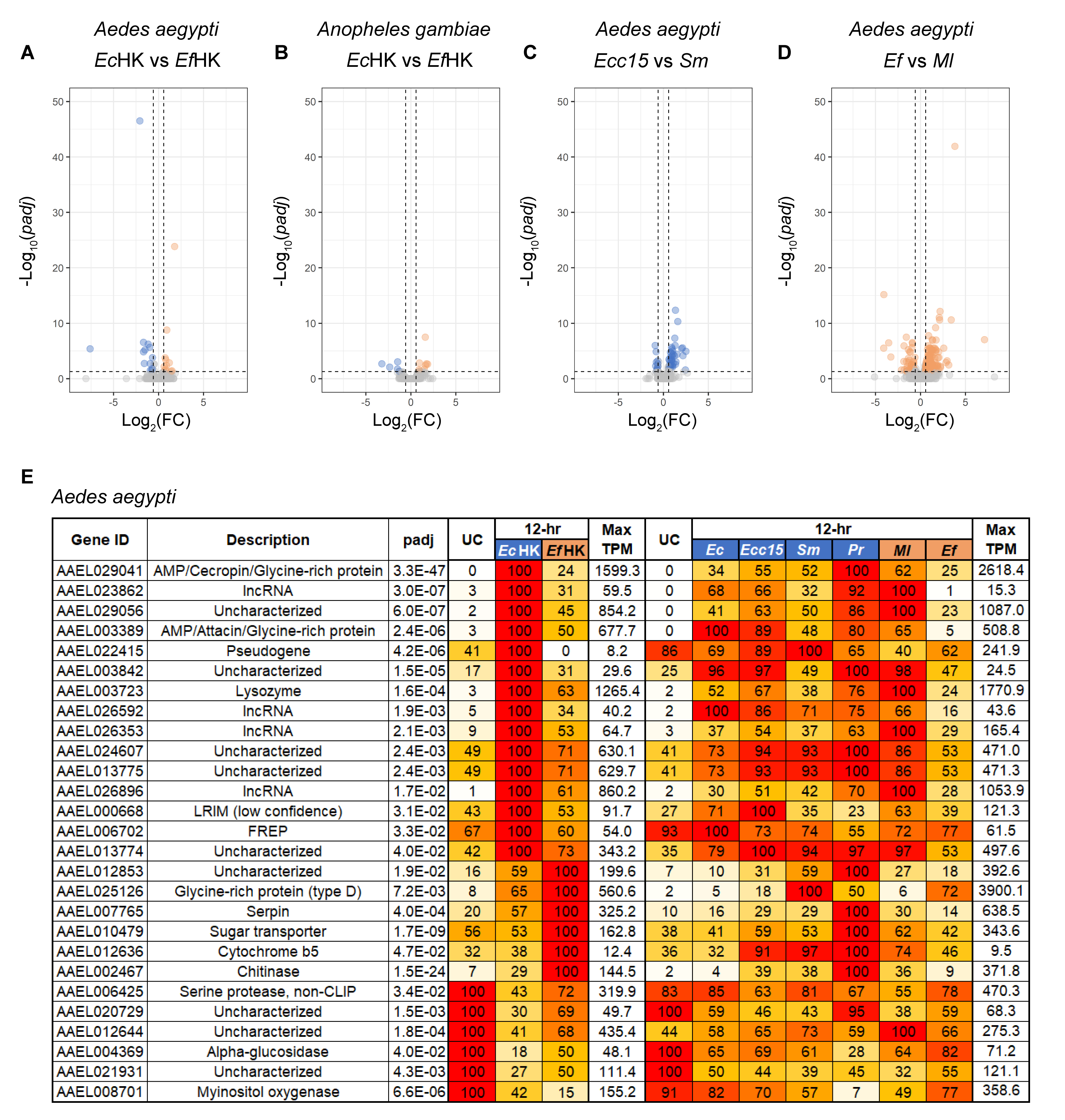

Supplement: Supplementary file 10 — Additional file 10: Fig. 4 S2. One-to-one comparisons of bacteria-challenged transcriptomes fail to demonstrate Gram type-specific transcriptional responses. Volcano plots depicting genes that are differentially expressed (DESeq2 fold-change ≥1.5, padj < 0.05) in a comparison of heat-killed Escherichia coli (EcHK) versus heat-killed Enterococcus faecalis (EcHK) (OD600 = 100) in Aedes aegypti (A) and Anopheles gambiae (B); further volcano plots compare the transcriptomes of Ae. aegypti challenged with live (3000 CFU) Erwinia carotovora carotovora 15 (Ecc15) versus Serratia marcescens type strain (Sm) (C) and Enterococcus faecalis versus (Ef) Micrococcus luteus (Ml) (D). (E) Table comparing TPMs of differentially expressed genes from panel (A) in live and heat-killed challenged conditions in Ae. aegypti. Conditions on the left-hand side include unchallenged mosquitoes (UC) and mosquitoes challenged with heat-killed Ec (EcHK), heat-killed Ef (EfHK) (OD600 = 100); all data are from the second RNAseq experiment. The right-hand side compares unchallenged (UC), and challenged with live Escherichia coli (Ec), Erwinia carotovora carotovora 15 (Ecc15), Serratia marcescens type strain (Sm), Providencia rettgeri (Pr), Micrococcus luteus (Ml), and Enterococcus faecalis (Ef). Within each comparison data are scaled to show relative expression so that the condition with the highest expression (shown in the max TPM column) is scored at ‘100’ and all lower expression values are expressed as a percentage of 100. [file 12864_2024_10153_MOESM10_ESM.tif]

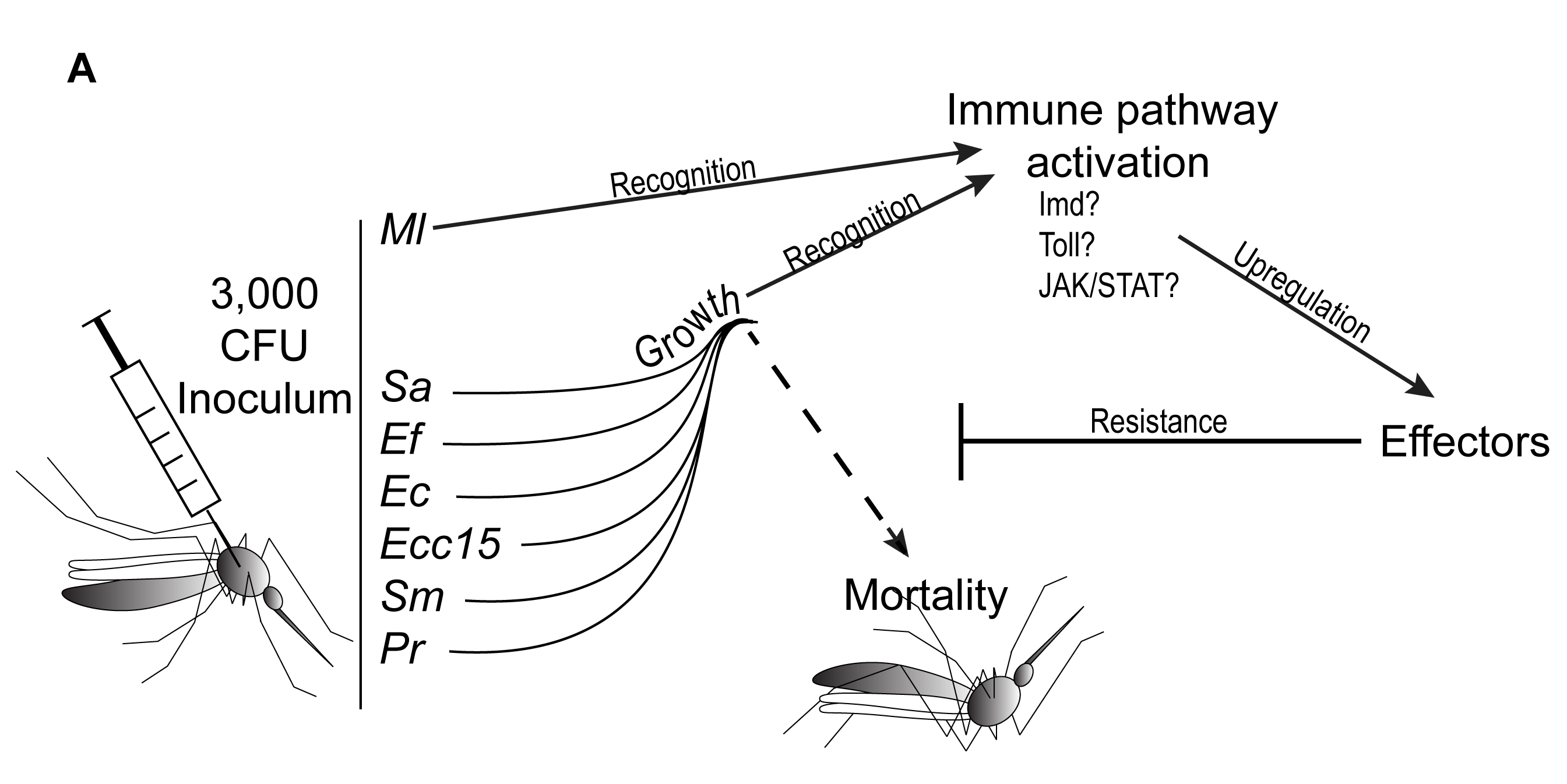

Supplement: Supplementary file 11 — Additional file 11: Fig. 5 S1. A scheme of the proposed interactions between bacterial pathogens, immune pathways, effector expression and mortality in challenged mosquitoes. [file 12864_2024_10153_MOESM11_ESM.tif]

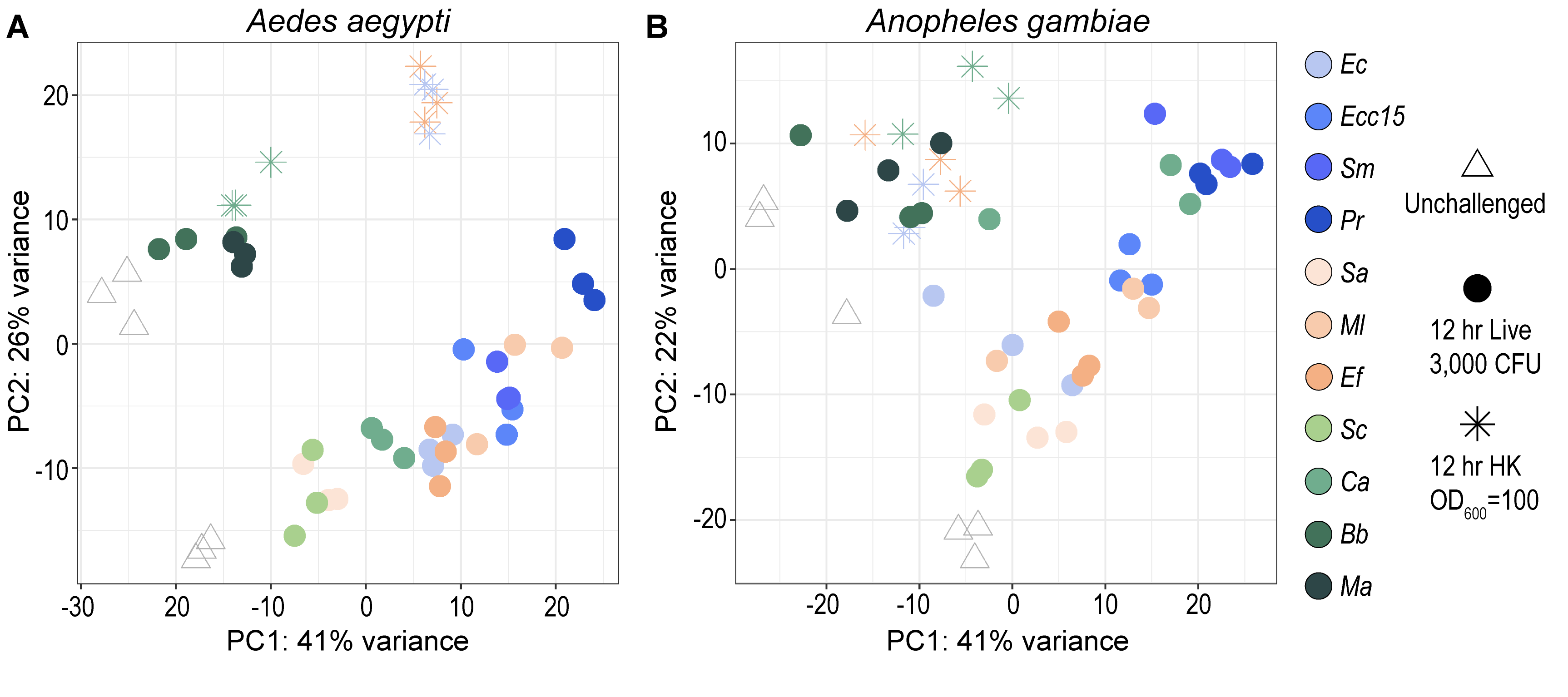

Supplement: Supplementary file 12 — Additional file 12: Fig. 6 S1. Principal component analyses combining transcriptomic data from two experiments in Aedes aegypti and Anopheles gambiae. Principal component analyses (PC1 vs PC2) of transcriptomes from Ae. aegypti (A) and Anopheles gambiae (s.l.) (B) combining data from two separate RNAseq experiments. From experiment #1: unchallenged (UC) mosquitoes and mosquitoes challenged with Escherichia coli (Ec), Erwinia carotovora carotovora 15 (Ecc15), Serratia marcescens type strain (Sm), Providencia rettgeri (Pr), Staphylococcus aureus (Sa), Micrococcus luteus (Ml), Enterococcus faecalis (Ef), Saccharomyces cerevisiae (Sc), and Candida albicans (Ca). From experiment #2: unchallenged (UC) mosquitoes, and mosquitoes challenged with Beauveria bassiana (Bb), Metarhizium anisopliae (Ma), and concentrated (OD600 = 100) heat-killed (HK) Ec, Ef, and Ca; all data are from the 12-hour timepoint. [file 12864_2024_10153_MOESM12_ESM.tif]

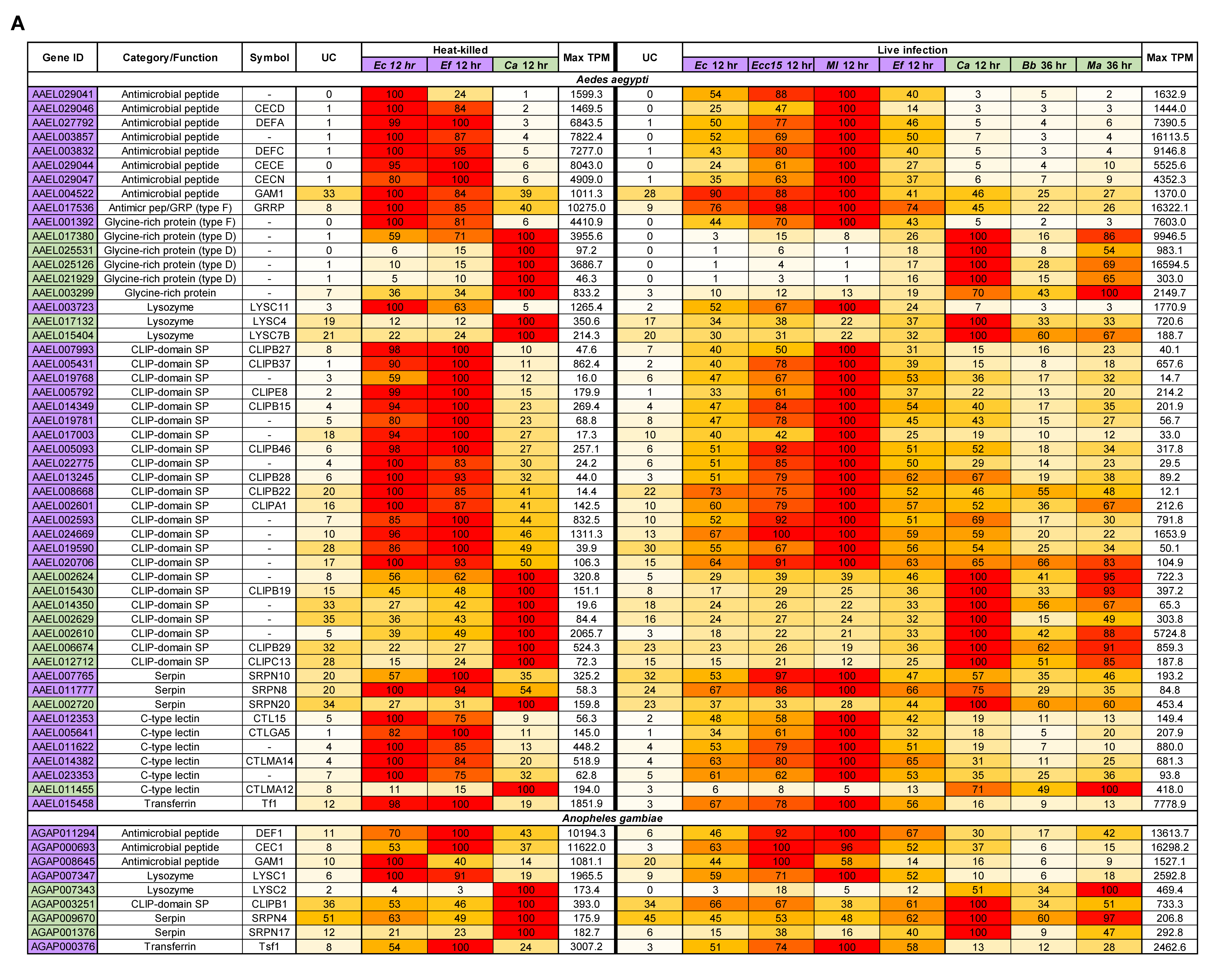

Supplement: Supplementary file 14 — Additional file 14: Fig. 6 S3. Selected genes in Aedes aegypti showing bacteria-specific or fungus-specific patterns of expression following heat-killed challenge or live infection. The left-hand side compares the expression of genes in unchallenged mosquitoes (UC) and mosquitoes challenged with heat-killed Ec (EcHK), heat-killed Ef (EfHK), or heat-killed Ca (CaHK); all data from the second RNAseq experiment. The right-hand side compares unchallenged (UC), and challenged with live Escherichia coli (Ec), Erwinia carotovora carotovora 15 (Ecc15), Serratia marcescens type strain (Sm), Providencia rettgeri (Pr), Micrococcus luteus (Ml), Enterococcus faecalis (Ef), Candida albicans (Ca) (from the first RNAseq experiment) and Beauveria bassiana (Bb) and Metarhizium anisopliae (Ma) (from the second RNAseq experiment). Within each comparison data are scaled to show relative expression so that the condition with the highest expression (shown in the max TPM column) is scored at ‘100’ and all lower expression values are expressed as a percentage of 100. Purple-labeled genes show greater responsiveness to bacterial challenges, while green-labeled genes show greater responsiveness to fungal challenges. [file 12864_2024_10153_MOESM14_ESM.tif]
